# The Importance of Early Steroid Use in IgA Vasculitis Complicated by Disseminated Intravascular Coagulation in an Adult

**DOI:** 10.7759/cureus.83628

**Published:** 2025-05-07

**Authors:** Kota Minami, Shun Yamashita, Sumika Uno, So Motomura, Shigehisa Aoki, Seiichi Kato, Masaki Tago

**Affiliations:** 1 Medical Training Center, Saga University Hospital, Saga, JPN; 2 Education and Research Center for Community Medicine, Faculty of Medicine, Saga University, Saga, JPN; 3 General Medicine, Saga University Hospital, Saga, JPN; 4 Cardiovascular Medicine, Saga University, Saga, JPN; 5 Pathology, Saga University, Saga, JPN; 6 Pathology and Microbiology, Saga University, Saga, JPN

**Keywords:** disseminated intravascular coagulation (dic), immunoglobulin a vasculitis, pathological findings, renal biopsy in nephrotic syndrome, steroid therapy

## Abstract

Adult patients with immunoglobulin A vasculitis (IgAV) may present with more severe renal symptoms than pediatric patients. Renal biopsy may be difficult to perform in patients with a high risk of bleeding, leading to delayed diagnosis and treatment, and poor prognosis. However, administering steroids before the diagnosis is confirmed may be avoided because of the possibility of the disappearance of the pathological findings of IgAV. A 22-year-old Japanese male was admitted to our hospital with abdominal pain extending from the epigastric to the umbilical region for 10 days, watery diarrhea, and rapidly progressive renal dysfunction. On admission, palpable purpura was observed on both palms and the dorsum of the feet. Blood and urine tests revealed disseminated intravascular coagulation (DIC) and nephrotic syndrome. Although continuous hemodiafiltration was initiated, renal function did not improve. Skin and duodenal biopsies performed for suspected IgAV failed to confirm the diagnosis, and renal biopsy was difficult to perform because of the high risk of bleeding. Steroid pulse therapy was initiated before a definitive diagnosis was made, and DIC began to improve. A renal biopsy performed on the 13th day after starting steroid therapy showed mesangial cell proliferation, and immunofluorescence showed IgA and C3 deposition, confirming IgAV. Steroids were gradually tapered, dialysis was discontinued, and the patient was discharged on the 62nd day of hospitalization. In cases of IgAV with DIC, it is necessary to prioritize steroid administration over renal biopsy, and the biopsy can be performed after the bleeding tendency improves.

## Introduction

Immunoglobulin A vasculitis (IgAV) is a systemic small-vessel vasculitis that is prevalent in pediatric populations, with an annual incidence of approximately 0.8-1.8 per 100,000 population in adults [1]. IgAV is associated with more severe renal dysfunction in adults than in children and is more likely to progress to chronic renal failure [2-4]. IgAV causes purpura, joint pain, abdominal pain, and renal impairment and is rarely a complication of disseminated intravascular coagulation (DIC) [2,5,6]. DIC is a condition in which infectious and non-infectious conditions, such as IgAV, cause dysregulation and excessive activation of blood coagulation due to the consumption of coagulation factors leading to a hypofibrinolytic system, resulting in disseminated microvascular thrombosis and bleeding tendency [7]. There have not been any previous reports on an adult IgAV case with DIC. In cases complicated by DIC, there is an increased risk of bleeding with invasive procedures, and renal biopsy can be difficult to perform [8]. Consequently, the inability to evaluate renal pathology may lead to a delayed or difficult diagnosis of IgAV, delaying the initiation of steroid treatment and worsening the renal prognosis [9,10]. Therefore, early diagnosis and treatment initiation are crucial for IgAV. However, steroid administration prior to the confirmation of the diagnosis can be avoided for several reasons. First, steroid administration may resolve inflammatory histopathological findings indicative of IgAV, such as mesangial hypercellularity and endocapillary proliferation, thus complicating the diagnosis [8]. Second, the recommended duration and tapering schedule of steroid therapy vary depending on the type of vasculitis. Without a confirmed diagnosis, premature tapering can lead to disease relapse, whereas prolonged administration can result in adverse events such as obesity, hypertension, and diabetes mellitus [11,12]. Finally, steroid administration could exacerbate the condition if the underlying disease is an infection [13,14]. In cases with atypical symptoms or complications, physicians may not be confident in their diagnosis of IgAV, and steroid administration before confirmation of the diagnosis may be avoided.

Here, we report a case of adult IgAV with DIC in which a renal biopsy was difficult to perform because of the high risk of bleeding. Although skin and gastrointestinal biopsies failed to establish the diagnosis, steroid administration prior to the diagnosis was unavoidable; the coagulation abnormalities improved, and the subsequent renal biopsy led to the final diagnosis of IgAV. Early steroid therapy is important in the management of IgAV with DIC.

## Case presentation

A 22-year-old Japanese man with a smoking history of four pack-years and a medical history of atopic dermatitis, bronchial asthma, and juvenile myoclonic epilepsy presented to his previous physician with persistent dull pain extending from the epigastric to the umbilical region and several episodes of non-bloody watery diarrhea per day for 10 days. His medications included valproic acid, fexofenadine, and mosapride citrate. The patient was diagnosed with acute enteritis and treated with fasting and sulbactam/cefoperazone. However, his abdominal pain and diarrhea did not improve, and his serum creatinine level rapidly increased to 4.8 mg/dL, prompting his transfer to our hospital.

**Figure 1:**
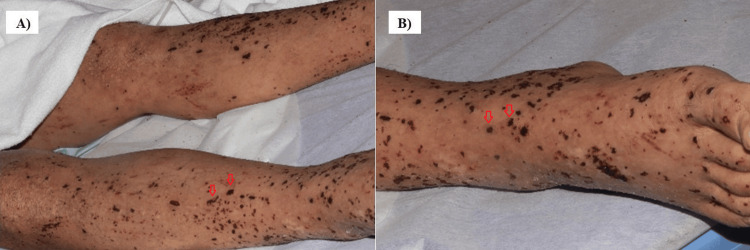
Skin findings of the lower extremities on admission. (A) Extensor surfaces of bilateral lower extremities, (B) dorsum of the right foot. Palpable purpura (arrows) was identified on the extensor surfaces of both lower legs, dorsum, and soles of both feet.

On admission, he was alert, with a body temperature of 38.0°C, heart rate of 121 beats/min, blood pressure of 134/74 mmHg, respiratory rate of 17 breaths/min, and oxygen saturation of 94% (on 1 L/min oxygen via a nasal cannula). Physical examination revealed pale conjunctiva, facial edema, abdominal distension, tenderness in the left upper abdomen and flank, painless enlargement of the right inguinal lymph node (33×16 mm), and palpable purpura on both palms, both lower legs, and the dorsum of the feet (Figures 1A, 1B). No conjunctival petechiae, dental caries, or heart murmur was observed. The laboratory findings on admission are shown in Tables 1, 2.

**Table 1:** Laboratory findings of complete blood count and coagulation tests on admission. MCV: mean corpuscular volume; PT-INR: prothrombin time-international normalized ratio; APTT: activated partial thromboplastin time; FDP: fibrinogen/fibrin degradation products

Laboratory items	Values	Units	Reference range
White blood cell	54.2	×10^3^/µL	3.3-8.6
Neutrophil	85	%	36.0-69.5
Red blood cell	2.34	×10^6^/µL	4.35-5.55
Hemoglobin	7.2	g/dL	13.7-16.8
MCV	91.9	fL	83.6-98.2
Hematocrit	21.5	%	40.7-50.1
Platelet	25.6	×10^4^/µL	15.8-34.8
PT-INR	1.52	-	0.90-1.10
APTT	65.3	s	25.0-40.0
Fibrinogen	434	mg/dL	200-400
FDP	30.7	µg/dL	0.0-5.0
D-dimer	11.9	µg/dL	0.0-1.0

**Table 2:** Laboratory findings of biochemical tests on admission. BUN: blood urea nitrogen; AST: aspartate aminotransferase; ALT: alanine aminotransferase; LDH: lactate dehydrogenase; γ-GTP: γ-glutamyl transpeptidase; CK: creatine kinase; CRP: C-reactive protein; ANA: antinuclear antibodies; ESR: erythrocyte sedimentation rate; PR3-ANCA: proteinase 3 antineutrophil cytoplasmic antibody; MPO-ANCA: myeloperoxidase-antineutrophil cytoplasmic antibody

Laboratory items	Values	Units	Reference range	Laboratory items	Values	Units	Reference range
Total protein	2.9	g/dL	6.6-8.1	Sodium	139	mEq/L	138-145
Albumin	0.6	g/dL	4.1-5.1	Potassium	4.4	mEq/L	3.6-4.8
BUN	40.6	mg/dL	8-20	Chloride	108	mEq/L	101-108
Creatinine	5.48	mg/dL	0.65-1.07	Immunoglobulin-G	561	mg/dL	861-1747
Total bilirubin	0.3	mg/dL	0.4-1.5	Immunoglobulin-A	312	mg/dL	93-393
Glucose	96	mg/dL	73-109	Immunoglobulin-M	23	mg/dL	33-183
CRP	21.4	mg/dL	0.00-0.14	C3	73	mg/dL	73-138
AST	33	U/L	13-30	C4	27	mg/dL	11-31
ALT	22	U/L	10-42	CH50	54	mmHg	31.6-57.6
LDH	655	U/L	124-222	ANA	<40	Times	<40
γ-GTP	118	U/L	13-64	ESR	30	mm/h	2-15
Amylase	80	U/L	44-132	Ferritin	1276	ng/mL	40-465
CK	27	U/L	59-248	PR3-, MPO-ANCA	<1.0	IU/mL	<3.5

**Figure 2:**
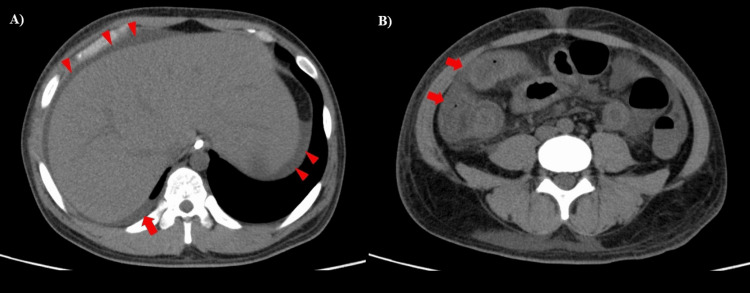
Chest and abdominal computed tomography without contrast enhancement on admission. Chest and abdominal computed tomography without contrast enhancement on admission showed a small amount of right pleural effusion (A, arrow), perihepatic ascites (A, arrowheads), and hepatomegaly. In addition, a thickened intestinal wall extending from the duodenum to the small intestine and ascending colon with increased surrounding fat density was observed (B, arrows).

Blood tests showed a white blood cell count of 54.2×10³/µL (neutrophil percentage of 85%), serum hemoglobin concentration of 7.2 g/dL, mean corpuscular volume of 91.9 fL, serum platelet count of 25.6×10⁴/µL, and serum C-reactive protein (CRP) concentration of 21.4 mg/dL. Coagulation abnormalities were also observed, including a prothrombin time-international normalized ratio of 1.5, an activated partial thromboplastin time of 65.3 seconds, a serum fibrinogen concentration of 434 mg/dL, a serum fibrin/fibrinogen degradation product (FDP) concentration of 30.7 µg/dL, and a D-dimer concentration of 11.9 µg/dL. He had a score of 4 on the Japanese Association for Acute Medicine DIC scoring system and was diagnosed with DIC complications [15]. In addition, he was complicated by nephrotic syndrome with a serum total protein concentration of 2.9 g/dL, a serum albumin concentration of 0.6 g/dL, and significant proteinuria (urine protein to creatinine ratio of 11 g/gCr) without hematuria. The hemolytic uremic syndrome, thrombotic thrombocytopenic purpura, sepsis, systemic lupus erythematosus, and vasculitis, including IgAV, were considered as differential diagnoses. Although the hemolytic uremic syndrome and thrombotic thrombocytopenic purpura were suspected because of an elevated serum creatinine concentration of 5.5 mg/dL and an elevated serum lactate dehydrogenase concentration (655 U/L), the serum total bilirubin and haptoglobin levels were normal. The stool samples were negative for O antigens and DNA polymerase chain reaction of pathogenic *Escherichia coli*. In addition, blood culture samples showed negative results. Tests for hypocomplementemia, antinuclear antibodies, proteinase 3 antineutrophil cytoplasmic antibodies, and myeloperoxidase antineutrophil cytoplasmic antibodies were all negative. Chest radiography performed on admission revealed no abnormalities. Chest and abdominal computed tomography scans without contrast enhancement revealed mild right pleural effusion, hepatomegaly, thickened intestinal walls, and ascites (Figures 2A, 2B).

**Figure 3:**
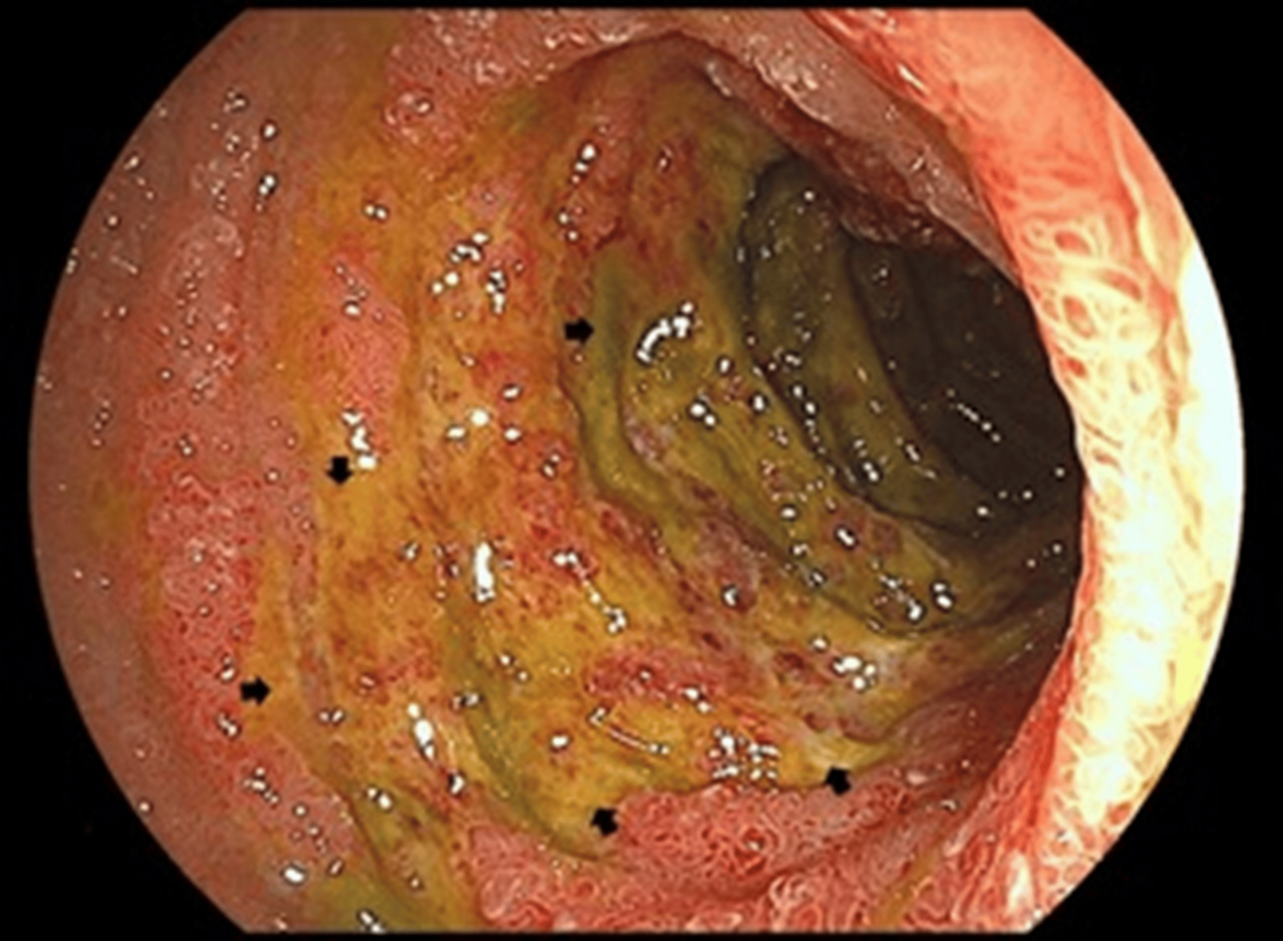
Findings of esophagogastroduodenoscopy. Diffuse map-like ulcers (arrows) were observed mainly in the duodenal bulb.

Considering the patient’s young age, presence of palpable purpura, progressive renal dysfunction with nephrotic syndrome, and findings suggestive of glomerulonephritis, IgAV was suspected. Although continuous hemodiafiltration was initiated immediately after admission, the renal function did not improve. The platelet count decreased to 6.8×10⁴/µL on the third hospital day, and the CRP and FDP concentrations increased to 26.7 mg/dL and 22.2 µg/mL, respectively, on the 10th hospital day. Esophagogastroduodenoscopy performed on the seventh day of hospitalization revealed extensive map-like ulcers, mainly in the duodenal bulb (Figure 3).

**Figure 4:**
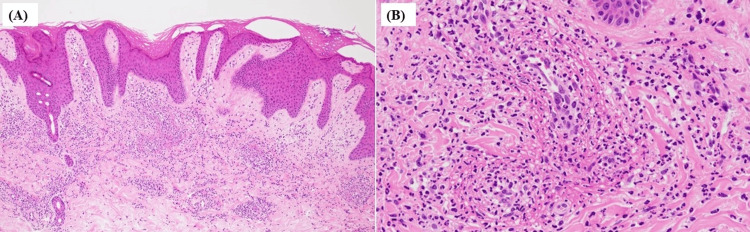
Pathological findings of purpura biopsy on the dorsum of the feet. (A) Hematoxylin and eosin staining (10×), (B) hematoxylin and eosin staining (40×). Infiltration of neutrophil-dominated inflammatory cells was observed around the superficial dermal vessels, along with nuclear debris, fibrin deposition, and extravasation of red blood cells. These findings were suggestive of leukocytoclastic vasculitis.

Pathological examination of the biopsied tissue from the duodenal ulcers revealed only neutrophil infiltration and tissue necrosis, without any findings indicative of vasculitis. Skin biopsy of the purpura on the dorsum of the feet performed on the 13th day of hospitalization revealed leukocytoclastic vasculitis, including perivascular neutrophil-dominant inflammation, nuclear debris, fibrin deposition, and extravasation of red blood cells without IgA deposition (Figures 4A, 4B).

**Figure 5:**
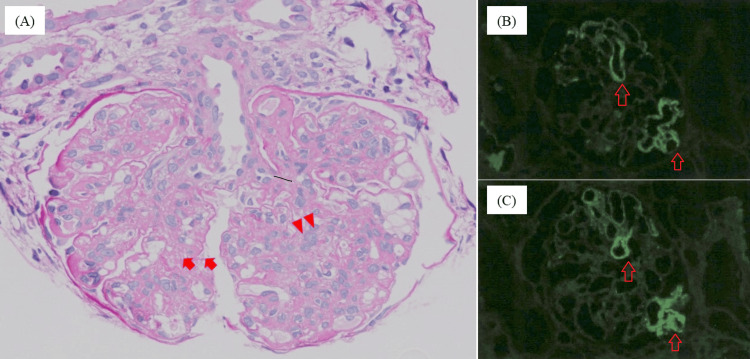
Pathological findings of renal biopsy. (A) Periodic acid-Schiff staining (40×), (B) immunofluorescence method (IgA), and (C) immunofluorescence method (C3). Periodic acid-Schiff staining showed mesangial matrix expansion (A, arrows), subendothelial edema, and endothelial cell proliferation (A, arrowheads). Immunofluorescence revealed IgA (B, arrows) and C3 (C, arrows) deposition in the mesangial area.

Although renal biopsy was necessary for the definitive diagnosis of IgAV and estimation of renal prognosis, it could not be performed due to the high risk of bleeding associated with DIC. Therefore, on the eighth hospital day, intravascular methylprednisolone at 500 mg/day for three days was initiated, followed by continued administration of 125 mg/day. The platelet count, serum FDP concentration, and serum CRP concentration improved on the 12th day of hospitalization. Chest and abdominal computed tomography without contrast enhancement on the 17th day of hospitalization showed resolution of ascites and intestinal wall thickening. On the 20th hospital day, methylprednisolone was replaced with oral prednisolone (1 mg/kg/day). On the 21st hospital day, the platelet count had normalized (16×10⁴/µL) and serum FDP concentration had improved (36.5 µg/mL), allowing a renal biopsy to be performed. Histopathological examination revealed mesangial matrix and endothelial cell proliferation on periodic acid-Schiff staining, and IgA and C3 deposition in the mesangial region on immunofluorescence staining, confirming the diagnosis of IgA vasculitis (Figures 5A-5C).

Prednisolone was tapered by 10 mg every seven days. Renal function improved, and hemodialysis was discontinued on the 47th day of hospitalization. After the resolution of abdominal pain and diarrhea, the patient was discharged on the 62nd day of hospitalization.

## Discussion

IgAV is a systemic small-vessel vasculitis prevalent in children [1]. Compared with pediatric cases, adult IgAV is reported to present with severe renal dysfunction [2,4]. The present case involved an adult with IgAV with a rare complication of DIC. Although IgA vasculitis was strongly suspected upon admission, skin and gastrointestinal biopsies failed to provide a definitive diagnosis. Because of the high risk of bleeding associated with DIC, renal biopsy could not be performed, and it was difficult to decide whether steroids should be administered prior to confirmation of the diagnosis. The present case suggests the importance of early steroid administration in cases with a high suspicion of IgA vasculitis and a high bleeding risk that leads to difficulties in performing a renal biopsy.

The main problem in the present case was the complication of DIC. Although histopathological examination by biopsy is essential for the diagnosis of IgAV, DIC complicates invasive procedures. Sites commonly biopsied for IgAV infection are the skin, gastrointestinal tract, and kidneys [16]. Among these, renal biopsy is important for estimating renal prognosis and confirming diagnosis [17]. However, renal biopsy is a highly invasive procedure with complications such as perirenal hematoma (1.3-33.3%) and gross hematuria (0.4-16.4%) [17]. In addition, transfusion, transcatheter arterial embolization, and surgical hemostasis or nephrectomy are required in 0.1-1.8%, 0.1-0.7%, and 0.01-0.2% of patients, respectively [17]. It is believed that these risks are significantly increased in patients at high risk of bleeding due to DIC. Systemic administration of steroids prior to the confirmation of the diagnosis is necessary to improve DIC and safely perform renal biopsies.

Another problem is the advantages and disadvantages of steroid administration before confirming the diagnosis with renal biopsy. Although treatment of the underlying disease with steroids can improve DIC and allow for renal biopsy, histopathological findings indicative of IgAV may also improve, making it difficult to confirm the diagnosis. However, the detection rates of key renal histopathological findings in IgAV, including mesangial hypercellularity, endocapillary proliferation, segmental glomerulosclerosis, interstitial fibrosis, tubular atrophy, and cellular crescents, showed no significant differences before and five years after steroid therapy: 28% vs. 30%, 60% vs. 49%, 95% vs. 84%, 35% vs. 47%, and 33% vs. 23%, respectively [8]. It has also been reported that interstitial fibrosis and tubular atrophy of the kidney persisted in all cases six months after steroid administration, whereas endocapillary proliferation and cellular crescents disappeared [18]. Furthermore, IgA and C3 deposition in glomeruli, which are critical for the diagnosis of IgAV, remained detectable in the majority of cases 16.2±9.3 months after steroid administration and in 78.2% of cases even after two years [19,20]. These findings suggested that steroid administration is unlikely to significantly affect the histopathological diagnosis of IgAV. In the present case, delaying the start of treatment could have led to a severe exacerbation of life and renal prognosis, and steroid administration was initiated before confirmation of the diagnosis. As a result, a renal biopsy was safely performed after improvement in DIC, and the patient was diagnosed with IgAV. In cases where a renal biopsy cannot be performed because of the high bleeding risk associated with DIC despite strongly suspected IgAV, it is necessary to administer steroids without hesitation before confirming the diagnosis and performing a renal biopsy after the improvement of DIC.

## Conclusions

This study discussed an adult-onset of IgAV complicated by DIC. Due to the risk of bleeding, we initially avoided performing a renal biopsy and were uncertain about administering steroids before a definitive diagnosis. As a result, DIC improved with steroid therapy, and a subsequent renal biopsy led to the diagnosis of IgAV. Steroid administration may not affect the histopathological findings of IgAV. Therefore, in adult cases of suspected IgAV with DIC, it is necessary to start steroid administration before confirming the diagnosis and to safely perform a renal biopsy after the risk of bleeding has decreased.
